# Additive‐Free Ti_3_C_2_T_x_ MXene Actuator with Large Deformation, Programmability, and High‐Humidity Stability via Precise Interlayer Spacing Control Engineering

**DOI:** 10.1002/advs.202510243

**Published:** 2025-08-25

**Authors:** Haowen Zheng, Liangliang Xu, Qian Yan, Zonglin Liu, He Chen, Huanxin Lian, Yunxiang Chen, Teng Fei, Yiming Hu, Fuhua Xue, Xu Zhao, Cong Zhang, Qingyu Peng, Xiaodong He

**Affiliations:** ^1^ National Key Laboratory of Science and Technology on Advanced Composites in Special Environments Center for Composite Materials and Structures Harbin Institute of Technology Harbin 150080 P. R. China; ^2^ Suzhou Research Institute of HIT Suzhou 215104 P. R. China; ^3^ Department of Ultrasound The First Hospital Harbin Medical University Harbin 150080 P. R. China

**Keywords:** additive‐free, interlayer spacing regulation, MXene, programmable actuation, soft actuator

## Abstract

Introducing external substances to intercalate MXene (Ti_3_C_2_T_x_) or combining MXene with other inert materials to construct bilayer/multilayer structures is the current mainstream solution for improving actuation performance of MXene‐based actuators. Possible issues include the degradation in mechanical and electrical properties of MXene, or the decrease in actuation performance or even structural damage of the actuator under frequent actuation. Besides, the structural and actuation performance stability of MXene‐based actuators under high humidity environment also remain challenges. These issues limit the potential multifunctional integration and sustainable applications of MXene‐based actuators. Here, an additive‐free Ti_3_C_2_T_x_ MXene actuator with multistimulus response, large deformation, programmability, and excellent stability under high humidity environment is fabricated. By sequentially assembling MXene nanosheets with significant size differences, additive‐free MXene film with gradient structure is obtained. An innovative cyclic low‐temperature annealing‐rehydration technology is proposed, which achieves precise control of interlayer *d*‐spacing, initial shape, and actuation behavior of the actuator, and significantly improves its structural and actuation performance stability under high humidity environment. This work not only provides a new paradigm for designing high‐performance MXene‐based actuators, but also deepens the fundamental understanding of interlayer engineering of 2D materials, laying the foundation for the development of next‐generation sustainable intelligent materials and devices.

## Graphical abstract

By sequentially assembling MXene nanosheets with significant size differences, a multistimulus responsive additive‐free MXene actuator with gradient structure is obtained. An innovative cyclic low‐temperature annealing‐rehydration technology is proposed, which achieves precise control of interlayer *d*‐spacing, initial shape, and actuation behavior of the actuator, and significantly improves its structural and actuation performance stability under high humidity environment.

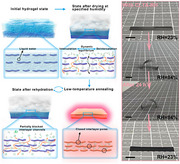

## 1 Introduction

Smart actuators that can perform complex deformation behaviors in response to external stimuli (e.g., heat,^[^ ^1^ ^]^ electric,^[^ ^2^ ^]^ light,^[^ ^3^, ^4^ ^]^ humidity,^[^ ^5^, ^6^ ^]^ magnetic,^[^ ^7^, ^8^ ^]^ chemical vapor,^[^ ^9^ ^]^ etc.) are attracting enormous attention in soft robotics,^[^ ^10^, ^11^ ^]^ wearable flexible electronics,^[^ ^11^ ^]^ human–machine interaction,^[^ ^11^ ^]^ and biomedical fields.^[^ ^12^, ^13^ ^]^ The actuation performance of soft actuators is highly dependent on the selection of materials and the design of the structures. Due to the tunable surface chemistry and excellent inherent optical, electrical, thermal, and mechanical properties, the 2D transition metal carbide, Ti_3_C_2_T_x_ MXene, has been proven to be one of the ideal materials for developing soft actuators.^[^ ^14^, ^15^, ^16^, ^17^, ^18^ ^]^ Compared with 1D fibers/yarns that can only realize linear or rotational actuation and 3D hydrogels/aerogels that need complex structure support, the 2D MXene films with unique layered structure composed of tightly stacked MXene nanosheets show significant advantages in soft actuators, such as high degree of freedom actuation behavior, rapid stimulus response caused by high specific surface area, ease of refinement and large‐scale manufacturing, and ease of conformity and integration with other materials, etc.^[^ ^19^, ^20^, ^21^, ^22^ ^]^ Although some good progress has been made in MXene‐based film actuators in recent years, there are still some challenges that need to be addressed.

The actuation performance improvement of most current MXene‐based film actuators relies heavily on additional additives in the material system.^[^ ^23^ ^]^ The nanoscale interlayer spaces of MXene films are vital channels for particle/ion transport.^[^ ^24^ ^]^ Under external stimulation, the diffusion of particles/ions in interlayer channels leads to changes in interlayer space and is transformed into macroscopic strain of MXene films. The existing strategies increase the interlayer spacing of MXene films through ion intercalation,^[^ ^25^, ^26^ ^]^ organic solvent intercalation,^[^ ^27^ ^]^ surface functional group modification,^[^ ^14^, ^28^ ^]^ polymer composites,^[^ ^17^, ^29^ ^]^ and other methods to enhance actuation performance. However, these methods typically involve complex chemical synthesis processes, and the use of organic solvents,^[^ ^30^, ^31^ ^]^ alkaline solutions,^[^ ^32^ ^]^ or ionic liquids^[^ ^33^ ^]^ for intercalation operations may cause environmental hazards and hinder sustainable development. More importantly, the introduction of external substances and residual intercalation agents often degrade the intrinsically outstanding mechanical and electrical properties of MXene films, which is highly detrimental to their potential multifunctional integration and applications. In addition to adjusting the interlayer spacing of MXene films to enhance their driving deformation ability, combining MXene film with other materials, such as polymer films, to form bilayer or multilayer structures, and utilizing the differential feedback of different materials on external stimuli to achieve efficient driving behavior, is another strategy to improve the actuation performance of MXene‐based film actuators.^[^ ^10^, ^34^ ^]^ However, due to the relatively weak interface bonding between heterogeneous materials, bilayer or multilayer structures may have poor interlayer stability under frequent actuations. Therefore, developing an additive‐free monolithic MXene film actuator with large driving deformation remains a challenge.

In addition to achieving large driving deformation of MXene film, it is also crucial to endow it with programmable actuation behavior. The current MXene‐based film actuators require complex chemical modifications or external templates to induce structural heterogeneity to achieve actuation behavior programmability.^[^ ^35^, ^36^, ^37^, ^38^, ^39^ ^]^ The key to this process actually lies mostly in the performance regulation of external additives, while MXene is only used as a stimulus responsive material, and its excellent mechanical properties have not been fully utilized. Therefore, achieving programmable actuation behavior of additive‐free monolithic MXene film is also a challenge. Besides, due to the high hydrophilicity of MXene, when MXene‐based film actuators are exposed to high humidity environments for a long time, the continuous intercalation of water molecules weakens the interlayer van der Waals forces, leading to structural collapse and decreased actuation performance.^[^ ^40^ ^]^ Therefore, developing MXene film actuator with long‐term stability under a high humidity condition is also crucial.

In this work, MXene nanosheets with significant size differences were synthesized and sequentially assembled to obtain additive‐free Ti_3_C_2_T_x_ MXene film with gradient structures. The structural stability of the additive‐free MXene film in high humidity environment has been significantly improved through an innovative cyclic low‐temperature annealing‐rehydration (CLTA‐RH) technology. By adjusting the ambient humidity during the CLTA‐RH process, precise control of the initial interlayer *d*‐spacing and initial curvature of MXene film, as well as programmable actuation behavior, have been achieved. Based on the asymmetric interlayer structure of large‐sized MXene layer and small‐sized MXene layer, and excellent photothermal/electrothermal conversion efficiency and high hydrophilicity of Ti_3_C_2_T_x_ MXene, this additive‐free MXene film can generate large (>520°, ΔRH = 61%; >580°, 835 mW cm^−2^; >630°, 2.5 V), stable, and controllable driving deformation behavior under the stimulation of humidity, light, and electricity. In addition, the MXene film exhibits excellent actuation behavior stability in high humidity environment (over 1500 cycles of light‐induced and electric‐induced stable driving deformation at 84% RH environment). This work provides a sustainable approach to regulate the interlayer spacing of MXene film without the need for chemical modification or high‐energy processes. It paves the way for the development of additive‐free MXene film actuators with large driving deformation, programmability, and stability under high humidity environment, which is expected to promote the development of MXene‐based actuators and related soft robotics towards an environmentally friendly and sustainable direction.

## 2 Results and Discussion

**Figure 1:**
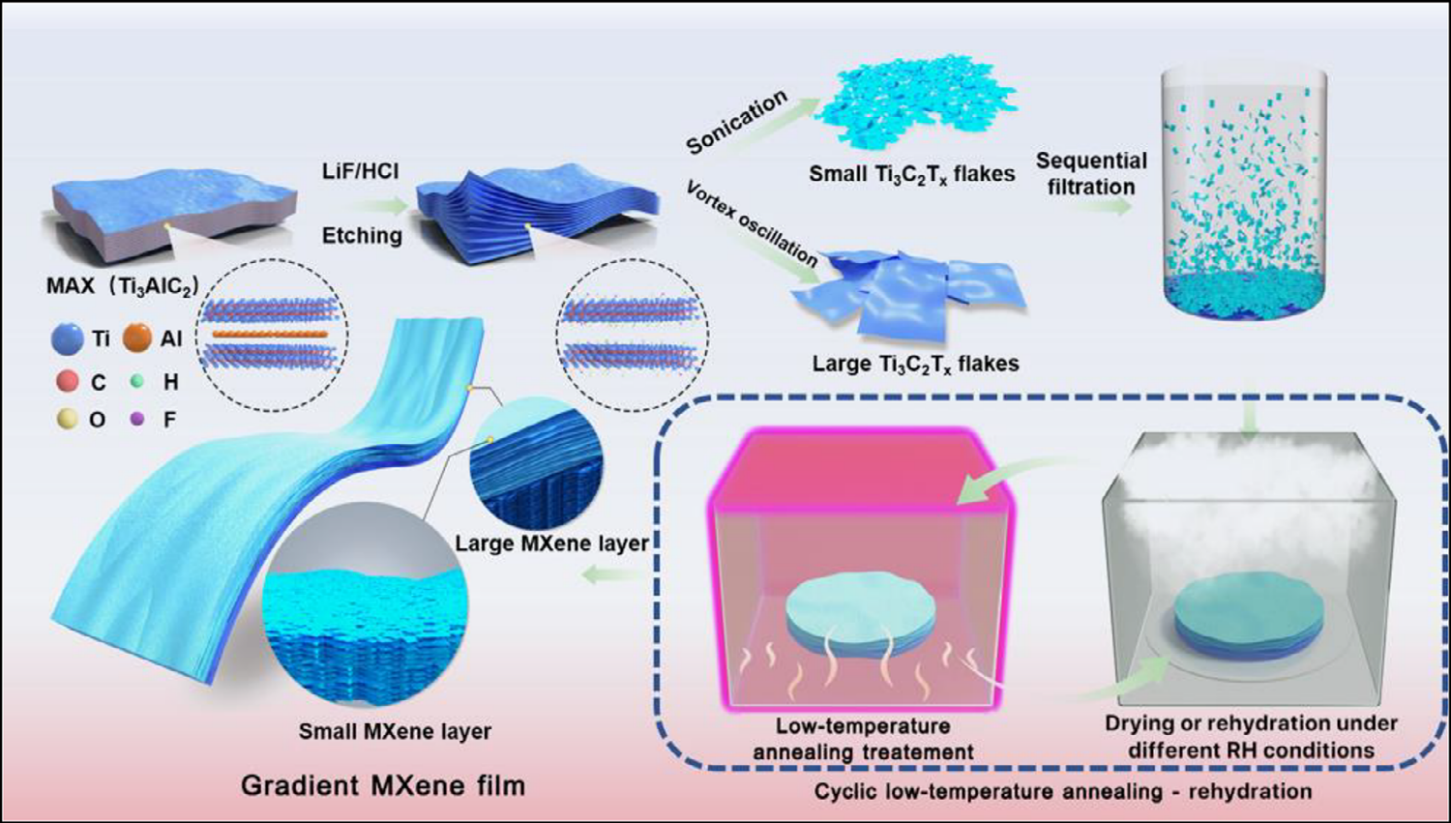
Schematic diagram of the fabrication procedure of the additive‐free Ti_3_C_2_T_x_ MXene film actuator.

**Figure** **1** schematically illustrates the fabrication process of the additive‐free Ti_3_C_2_T_x_ MXene film actuator. The Ti_3_C_2_T_x_ MXene nanosheets with different sizes were prepared using different synthesis processes, and then sequentially vacuum‐assisted filtration was performed on the synthesized MXene nanosheets with different sizes to obtain additive‐free MXene film with gradient structure. Finally, an innovative cyclic low‐temperature annealing‐rehydration process was employed to achieve programmable shaping of the MXene film and significantly improve its performance stability.

**Figure 2:**
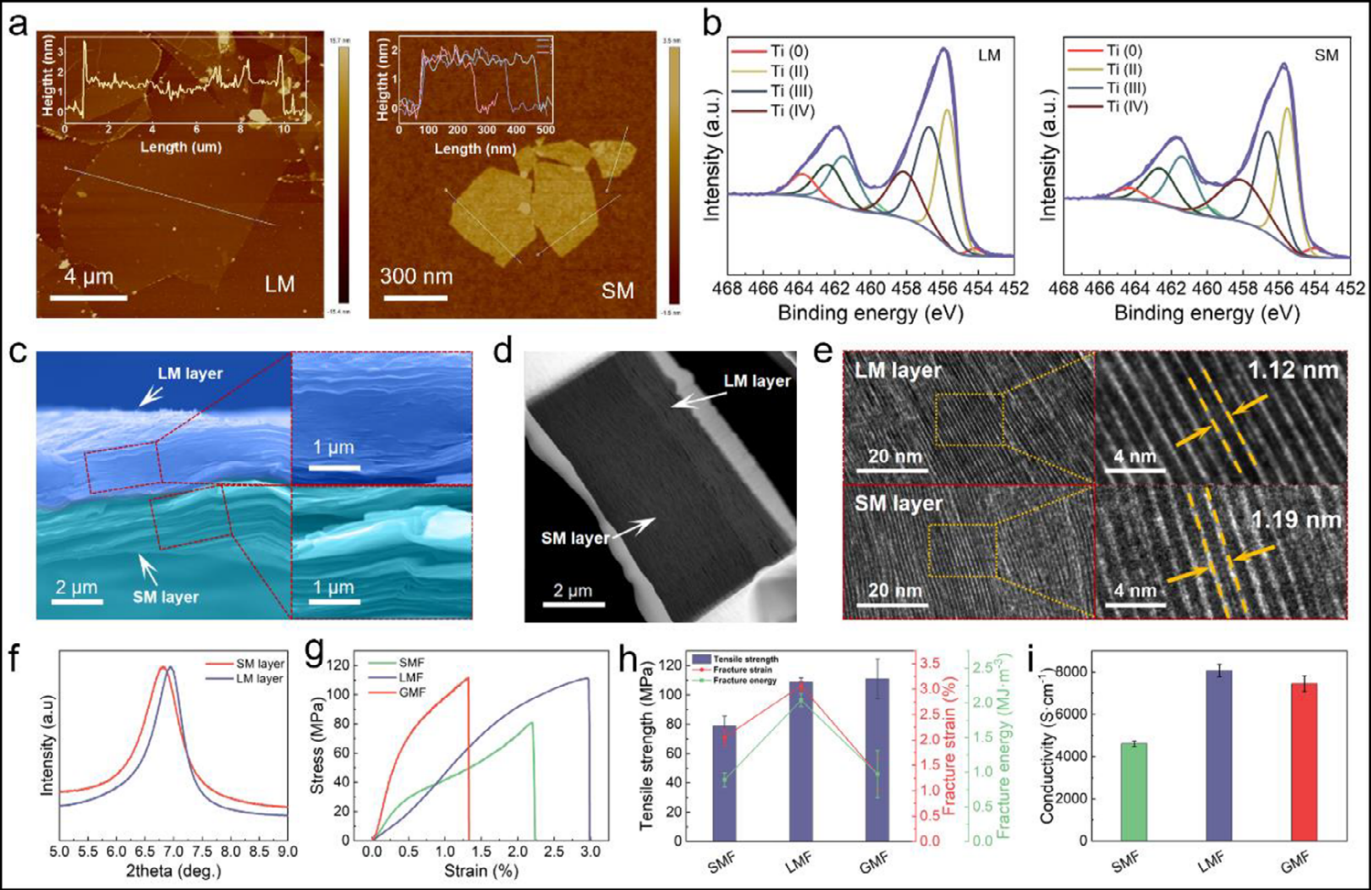
Characterization of the additive‐free Ti_3_C_2_T_x_ MXene film with gradient structure. a) AFM images of LM nanosheets and SM nanosheets. b) High‐resolution XPS spectra of LM and SM in Ti 2p region. c) Cross‐sectional SEM images of GMF and amplification of LM layer and SM layer. d) Cross‐sectional HADDF image of GMF. e) HRTEM images of LM layer and SM layer of GMF. f) XRD patterns of the SM layer and LM layer of GMF. e) Stress–strain curves of the SMF, LMF, and GMF. f) Comparison of tensile strength, fracture strain, and fracture energy of the SMF, LMF, and GMF. g) Comparison of electrical conductivity of the SMF, LMF, and GMF.

The controllable synthesis of clean and high‐quality Ti_3_C_2_T_x_ MXene nanosheets with significant size differences is the basis for constructing the additive‐free MXene film actuator with gradient structure. The specific synthesis process of the large‐sized and small‐sized Ti_3_C_2_T_x_ MXene nanosheets are given in the experimental section. LiF/HCl mixed solution was selected to etch the Al atom layer in Ti_3_AlC_2_ MAX phase. The reaction product was washed multiple times with dilute hydrochloric acid and deionized water to remove excess intercalating agents and obtain the multilayer Ti_3_C_2_T_x_ MXene dispersion. The etched multilayer MXene exhibits a classic accordion‐like structure (Figure S1, Supporting Information), indicating the high etching efficiency. Different delamination technologies were applied to multilayer MXene dispersions to obtain MXene nanosheets with different sizes. The large‐sized MXene (LM) nanosheets were obtained by vortex oscillation followed by centrifugal treatment of multilayer MXene dispersion, and the small‐sized MXene (SM) nanosheets were obtained by ultrasonic with Ar gas followed by centrifugal treatment of multilayer MXene dispersion. Vortex oscillation can usually generate high shear forces, promote water molecules to penetrate into the interlayer of multilayer MXene, weaken the interlayer interaction forces, and effectively achieve the delamination of multilayer MXene.^[^ ^41^ ^]^ Due to the lower energy input and mechanical vibration intensity, vortex oscillation has limited ability to break the lateral layers of multilayer MXene, thus enabling the acquisition of LM nanosheets. In contrast, the ultrasonic cavitation effect can generate high‐energy shock waves, which not only completely overcome interlayer forces to achieve the delamination of MXene nanosheets, but also break down the delaminated MXene nanosheets into smaller sized nanosheets.^[^ ^42^ ^]^ Atomic force microscopy (AFM) images show that the MXene nanosheets prepared by different synthesis processes exhibit typical 2D nanosheet structure with a thickness of ≈1.6 nm and show significant lateral size differences (**Figure** **2**a). The average lateral size of LM and SM nanosheets are ≈10.3 and ≈0.35 µm, respectively. The transmission electron microscope (TEM) images of the LM and SM nanosheets are also provided (Figure S2, Supporting Information). X‐ray photoelectron spectroscopy (XPS) was further characterized to analyze the chemical composition of the synthesized Ti_3_C_2_T_x_ MXene with different sizes (**Figure** **2**b; Figure S3, Supporting Information). The peak difference of Ti(IV) characteristic peak around 458 eV reflects the difference in oxidation degree of the MXene nanosheets with different sizes. The results indicate that the SM nanosheets have a higher oxygen content, and the reason is that compared to LM, the edges of the SM nanosheets obtained by ultrasonic treatment have more defects.^[^ ^43^ ^]^

Sequential vacuum‐assisted filtration process was used to fabricate the additive‐free MXene film with gradient structure. First, the LM suspension was subjected to vacuum‐assisted filtration until there is no visible water on the surface of the film. Then, the SM suspension was added and further filtered, and the MXene film with gradient structure was obtained. After that, the MXene film was transferred to a specific relative humidity (RH) environment for drying, and then was annealed the dried film at 60 °C. Subsequently, the MXene film was transferred again to the previous RH environment for rehydration. This process was repeated five times to obtain the additive‐free MXene film actuator with gradient structure. The CLTA‐RH process is of great significance for the configuration and stability (especially the structural stability and actuation performance stability in a high RH environment) improvement of the additive‐free MXene film, which we will elaborate on in the follow‐up content. Figure 2c shows the cross‐sectional scanning electron microscopy (SEM) image of the MXene film with gradient structure (gradient MXene film, GMF). Both the LM layer and the SM layer exhibit highly oriented in‐plane layered structures, and the consistency difference between LM layer and SM layer can be clearly observed, that is, the MXene nanosheets are stacked more densely in the LM layer and relatively loosely in the SM layer. In addition to the fact that the LM layer at the bottom bears greater pressure than the SM layer at the top during vacuum‐assisted filtration process, the presence of more oxygen‐containing functional groups of the SM nanosheets leads to greater electrostatic repulsion and adsorption of more water molecules, which is also an important reason why the stacked structure of the SM layer is looser than that of the LM layer.^[^ ^44^ ^]^ As a comparison, the cross‐sectional SEM images of the pure LM film (LMF) and pure SM film (SMF) are also provided (Figure S4, Supporting Information), and it is observed that the LMF is also denser than SMF. Focused ion beam (FIB) was used to cut the GMF to obtain an ultrathin sample, and its cross‐sectional microstructure were further observed using high‐angle annular dark‐field scanning transmission electron microscopy (HAADF‐STEM) and high‐resolution transmission electron microscopy (HRTEM). The cross‐sectional HAADF‐STEM image shows the distinct gradient structure of the GMF (Figure 2d), and the higher brightness of SM layer indicates its higher oxygen content than LM layer, which is consistent with the XPS results. The HRTEM images of LM and SM layers of GMF clearly display the lattice fringes of MXene nanosheets with spacings of 1.12 and 1.19 nm, respectively (Figure 2e). The results can also be attributed to the more abundant hydrophilic functional groups in SM nanosheets, which can provide more binding sites for water molecules and allow more intercalated water molecules to enter the interlayer structure. X‐ray diffraction (XRD) test was further characterized to demonstrate the gradient structure of GMF. The characteristic absorption peaks of the (002) crystal plane of the LM layer and SM layer are located at 6.94° and 6.81°, respectively (Figure 2f). According to the Bragg equation, the corresponding interlayer *d‐*spacing is calculated to be 1.29 and 1.35 nm, respectively. The *d*‐spacing values calculated according to the Bragg equation are larger than the results shown in HRTEM images, and the reason is that the instantaneous interlayer spacing of MXene nanosheets largely depends on the RH of the testing environment,^[^ ^14^ ^]^ therefore, the XRD testing conditions at a higher water molecule level (atmospheric conditions) determine the larger interlayer spacing results. Figure 2g gives the stress–strain curves of the SMF, LMF, and GMF (50 wt% LM and 50 wt% SM). Due to the denser stacking between LM nanosheets, the LMFs show higher tensile strength (108.8 ± 2.7 MPa), fracture strain (3.06 ± 0.09%), and fracture energy (2.04 ± 0.09 MJ m^−3^), which are 1.38, 1.49, and 2.31 times higher than SMFs, respectively (Figure 2h). The tensile strength of GMFs (111.8 ± 13.6 MPa) is comparable to LMFs, which can be attributed to the structural densification induced by the CLTA‐RH process (LMFs and SMFs have not undergone the CLTA‐RH treatment). However, it is precisely due to the stronger interactions between MXene nanosheets in the GMFs, the hindered interlayer sliding leads to their lower fracture strain (1.32 ± 0.31%) and fracture energy (0.97 ± 0.34 MJ m^−3^). In addition, the additive‐free MXene films exhibit impressive electrical conductivity (Figure 2i). The electrical conductivities of LMFs and SMFs are 8069 ± 297 and 4612 ± 129 S cm^−1^. The GMFs maintain the high conductivity of LMFs, reaching 7453 ± 376 S cm^−1^ (≈92.4% of LMFs), making GMFs highly promising for applications in electric‐driven actuators and related fields.

**Figure 3:**
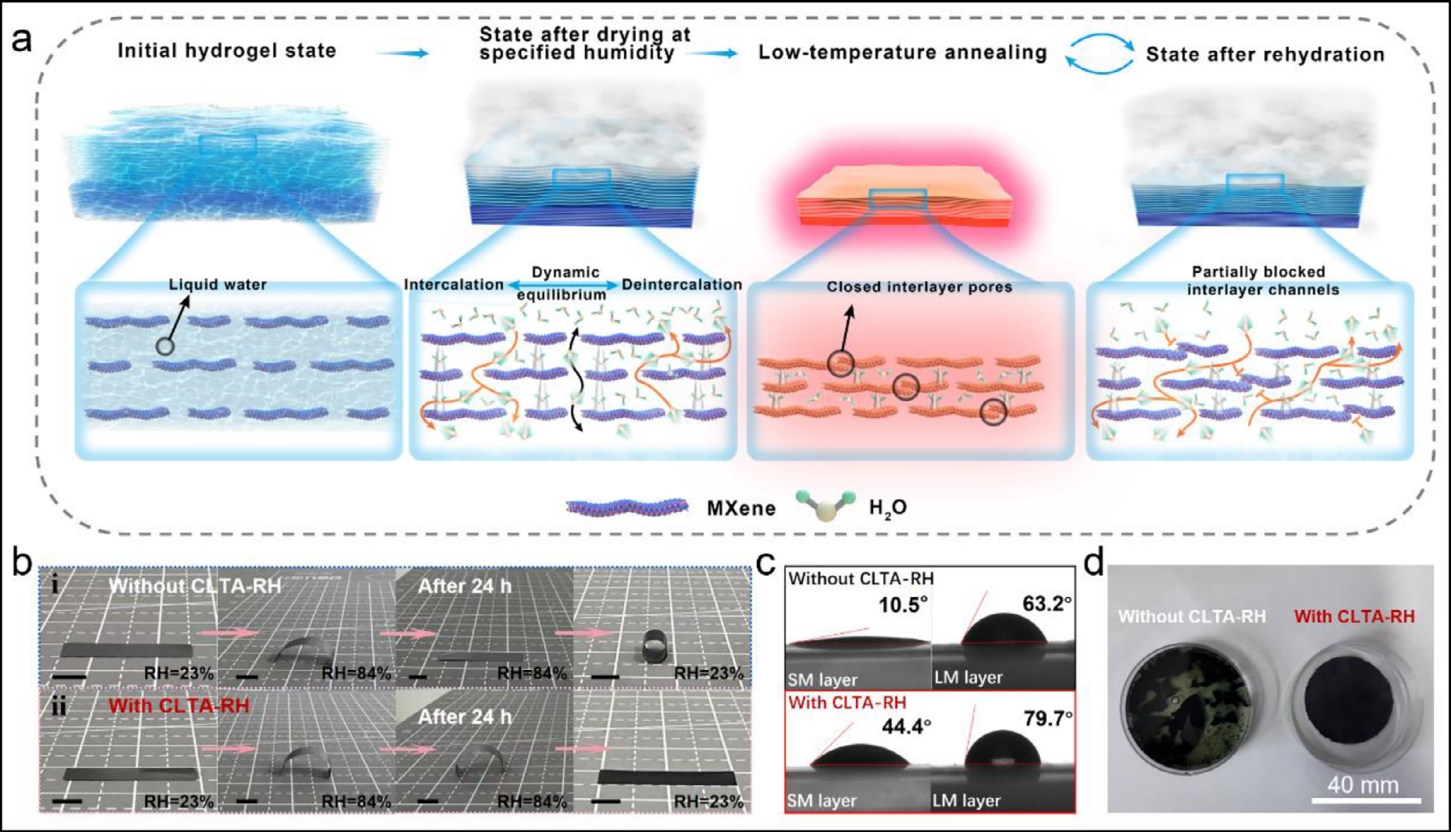
Mechanism analysis of the CLTA‐RH process. a) Schematic diagram of the CLTA‐RH process. b) Deformation stability of the GMF with and without the CLTA‐RH treatment under high humidity environment (84% RH). Scale bar = 5 mm. c) Comparison of the contact angles of the GMF with and without the CLTA‐RH treatment. d) Comparison of the water stability of the GMF with and without the CLTA‐RH process.

The CLTA‐RH treatment is crucial for regulating the performance of the GMF, and we systematically analyzed the process. As shown in **Figure** **3**a, the wet GMF can be regarded as a “hydrogel” state at the end of filtration, where the liquid water fills the interlayers of MXene, which helps to maintain the ordered layered structure of the film formed by negative pressure. The wet GMF is placed in a specific environment RH for natural drying, and the unrestricted “free water” inside the film can spontaneously move along the interlayer gaps to the surface of the film and diffuse into the environment. During this process, the capillary pressure caused by “free water” evaporation can promote the tight stacking of MXene layers, enhance their interlayer interactions, and lead to the structural densification of the hydrogel film. When the partial pressure of water vapor in GMF reaches equilibrium with the ambient humidity, evaporation of the “free water” stops and a dynamic equilibrium is established between the intercalation and deintercalation of water molecules on the film surface. At this stage, the “free water” transitions from the liquid phase to the vapor phase, enabling it to move freely between MXene interlayers or be captured by hydrophilic functional groups to collectively serve as the interlayer supports. During this process, the water molecules can be regarded as a “plasticizer” that can facilitate the slip of MXene nanosheets and eliminate the residual stresses generated during the filtration process.^[^ ^45^ ^]^ However, the GMF that has undergone only one routine drying treatment is still in an unstable state. The almost fully interconnected nanoscale interlayer channels allow water molecules to reintercalate or deintercalate, which leads to a decrease in actuation performance and even layer dissociation of the GMF under a high humidity condition, similar to most MXene‐based film actuators.^[^ ^40^ ^]^ As shown in Figure 3b–i, for a flat GMF naturally dried at a 23% RH environment without CLTA‐RH treatment, when it was placed in an 84% RH environment, the GMF rapidly bent and deformed towards the LM layer side (the actuation mechanism will be explained in the follow‐up content). However, the driving deformation of this GMF without CLTA‐RH treatment cannot remain stable. When the GMF was placed in the 84% RH environment for 24 h, its shape autonomously recovered to the flat state. When the GMF was placed back in a 23% RH environment, the GMF quickly bent and deformed towards the SM layer side, forming a curled structure.

The use of high‐temperature heat treatment to remove intercalation water has been proven to be an effective method for improving the hydration stability of MXene‐based films.^[^ ^46^ ^]^ However, excessively high temperatures (>200 °C) will cause the layers of MXene film to stack too tightly, creating a “dead space” that hinders the diffusion of water molecules between layers and makes the external water molecules more difficult to enter the interior of the film.^[^ ^42^ ^]^ These problems can greatly affect the actuation performance of the MXene‐based films. To solve the above problems, by reducing the annealing temperature (60 °C) and introducing a rehydration process, we have successfully achieved extraordinary structural and actuation stability of GMF under a high humidity condition. As shown in Figure 3b‐ii, for a flat GMF naturally dried at a 23% RH environment with CLTA‐RH treatment, when it was placed in an 84% RH environment, the GMF rapidly bent and deformed towards the LM layer side. Even after 24 h, this GMF remained in the bent state without any significant decrease in actuation performance. When the GMF was placed back in a 23% RH environment, it recovered to the flat state. The lower annealing temperature allows for the retention of bound water and some free water within GMF, while preventing the decomposition of hydrophilic functional groups. As low‐temperature annealing proceeds, the interlayer spacing of GMF decreases, but some water molecule diffusion channels are still retained. During the rehydration process, water molecules reintercalate into the interlayers of the GMF, eliminating residual stresses generated during annealing, and making the structure of the GMF more stable. The contact angles of GMFs with and without CLTA‐RH treatment were also characterized to analyze the effect of CLTA‐RH process on the hydrophilicity of GMF. As shown in Figure 3c, the SM layer and LM layer of GMF without CLTA‐RH treatment have asymmetric water contact angles of 10.5° and 63.2°, respectively. The SM layer is highly hydrophilic, and the water droplet can almost completely penetrate into its interior, indicating its smooth water transport channels. After CLTA‐RH treatment, the water contact angles of SM layer and LM layer of the GMF increased to 44.4° and 79.7°, respectively, but remained hydrophilic. In addition, the GMFs were placed in water and subjected to ultrasonic treatment. After 1 min ultrasonic treatment, the GMF without CLTA‐RH treatment dissociated in the water, while the GMF with CLTA‐RH treatment still maintained a stable structure (Figure 3d). The above results indicate that the CLTA‐RH treatment can significantly improve the structural stability and actuation performance stability of GMF, especially under a high humidity condition.

**Figure 4:**
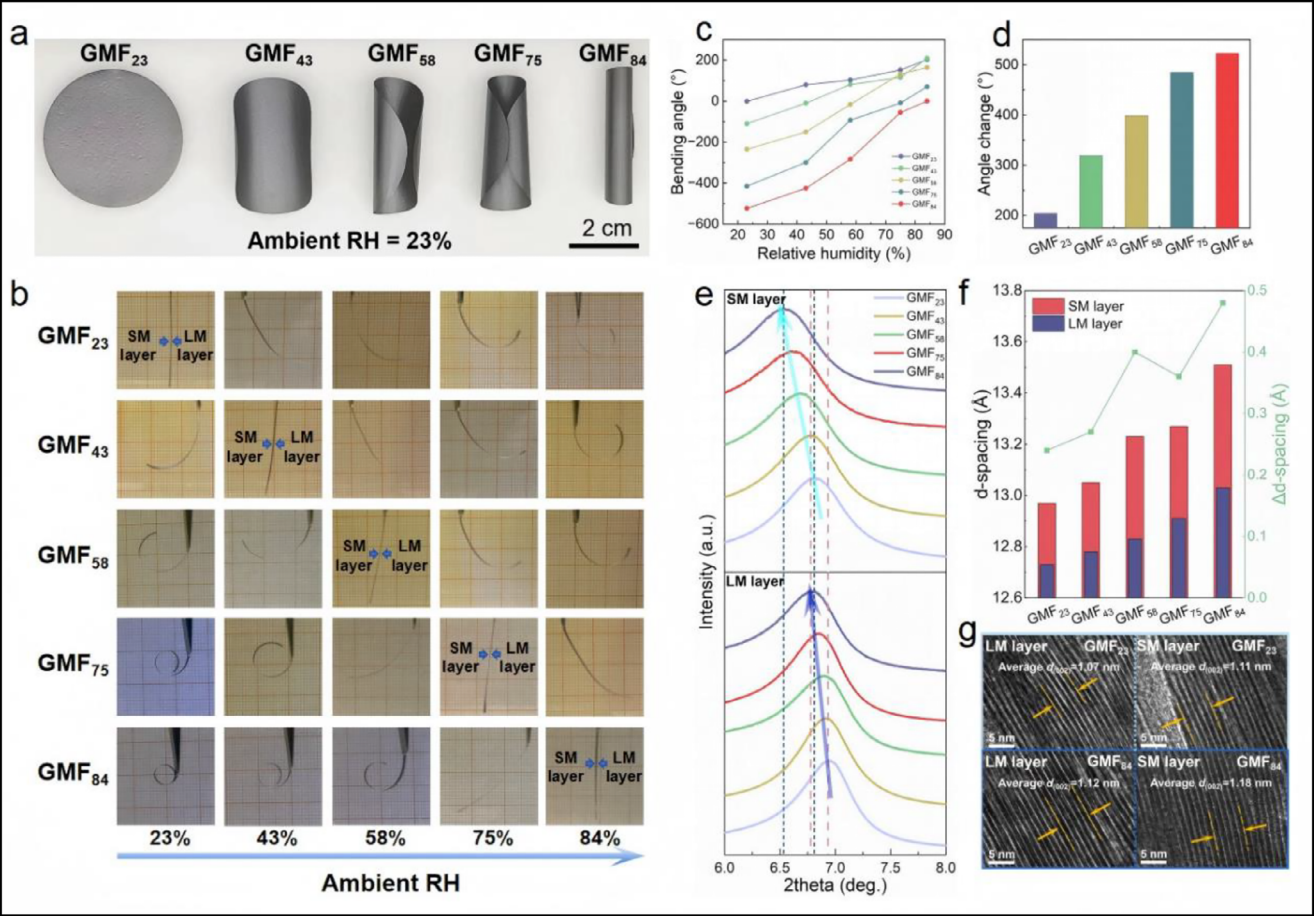
Programmability of the GMFs, including the programming of its interlayer *d*‐spacing, initial shape, and actuation behavior. a) The optical images of GMFs prepared by CLTA‐RH process with different RHs. b) Humidity responsive actuation behavior of the different GMF samples. c) Deformation angle of the different GMF samples under different RHs. d) The adjustable deformation range of the different GMF samples. e) XRD patterns of the SM layer and LM layer of different GMF samples. f) Interlayer *d*‐spacing of the SM layer and LM layer of different GMF samples, and the interlayer *d*‐spacing difference between SM layer and LM layer. g) HRTEM images of SM layers and LM layers of the GMF_23_ and GMF_84_.

The mechanism of CLTA‐RH process determines that the initial shape and deformation behavior of GMF under a specific humidity are highly related to the RH of the CLTA‐RH process. Because the GMF remains mechanically static throughout the entire CLTA‐RH process, the RH corresponding to its flat equilibrium state is the RH condition used in the CLTA‐RH process. Therefore, by regulating the RH of the CLTA‐RH process, the initial shape of GMF under atmospheric RH and the deformation under different ambient RHs can be adjusted. **Figure** **4**a gives the optical images of GMFs prepared by CLTA‐RH process with different RHs, where the ambient RH is 23% (laboratory RH in this work). The samples are named GMF_23_, GMF_43_, GMF_58_, GMF_75_, and GMF_84_, respectively, and the subscripts correspond to the RHs during the CLTA‐RH processes (23%, 43%, 58%, 75%, and 84%). It can be seen that the GMF_23_ is in a flat state at 23% ambient RH, while GMF_43_, GMF_58_, GMF_75_, and GMF_84_ are all in a curled state (the SM layer is on the inner side, and the LM layer is on the outer side). The higher RH of the CLTA‐RH process, the smaller initial curvature of the final obtained GMF sample under 23% ambient RH. Figure 4b shows the driving deformation of these GMF samples under different RHs, and it can be seen that as the ambient RH gradually increases, all GMF samples undergo driving deformation towards the LM layer side. As a comparison, the states of LMF and SMF at 23% RH and 84% RH are also provided (Figure S5, Supporting Information). The slight deformation may be due to the structural differences between the upper and lower surfaces of the MXene films, which is common in the thin films obtained by vacuum‐assisted filtration method. The deformation angle of the GMFs in a flat state is defined as 0°, and the deformation angles towards the LM layer side and SM layer side are defined as positive and negative values, respectively. The deformation angle changes of the samples under different RHs are given in Figure 4c. With the ambient RH increases, the deformation angle of the GMFs gradually increases. It is worth mentioning that there are significant differences in the humidity response deformation range of GMFs prepared under different RHs of CLTA‐RH process. When the ambient RH increases from 23% to 84%, the bending angle of GMF_23_ increases from the initial 0° to 203°, while the bending angle of GMF_84_ increases from the initial −523° to 0°. As shown in Figure 4d, the adjustable deformation range of GMF gradually increases with the increase of RH of the CLTA‐RH process. In order to analyze the relevant reasons, XRD characterization was performed on the LM and SM layers of GMFs prepared under different RHs of CLTA‐RH process. As shown in Figure 4e, as the RH of the CLTA‐RH process increases, the (002) diffraction peaks of LM layer and SM layer of GMFs shift significantly towards smaller angles, indicating the increase in interlayer *d*‐spacing. Figure 4f shows the specific values of interlayer *d*‐spacing. As the RH of the CLTA‐RH process increases from 23% to 84%, the *d*‐spacing of LM layer increases from 12.73 to 13.3 Å, and the *d*‐spacing of SM layer increases from 12.97 to 13.51 Å. The variation in interlayer *d*‐spacing of the SM layer (0.54 Å) is greater than that of the LM layer (0.30 Å), which is a result of the SM layer having a large initial interlayer *d*‐spacing that can accommodate more water molecules. More importantly, the interlayer *d*‐spacing difference between SM layer and LM layer (Δ*d*‐spacing) also increases with the increase of RH of the CLTA‐RH process, from 0.24 Å at 23% RH to 0.48 Å at 84% RH, which is consistent with the results of HRTEM (Figure 4g). A larger interlayer *d*‐spacing difference means greater asymmetric deformation of the two layers under stimulation, which endows the GMF with greater deformation range.

**Figure 5:**
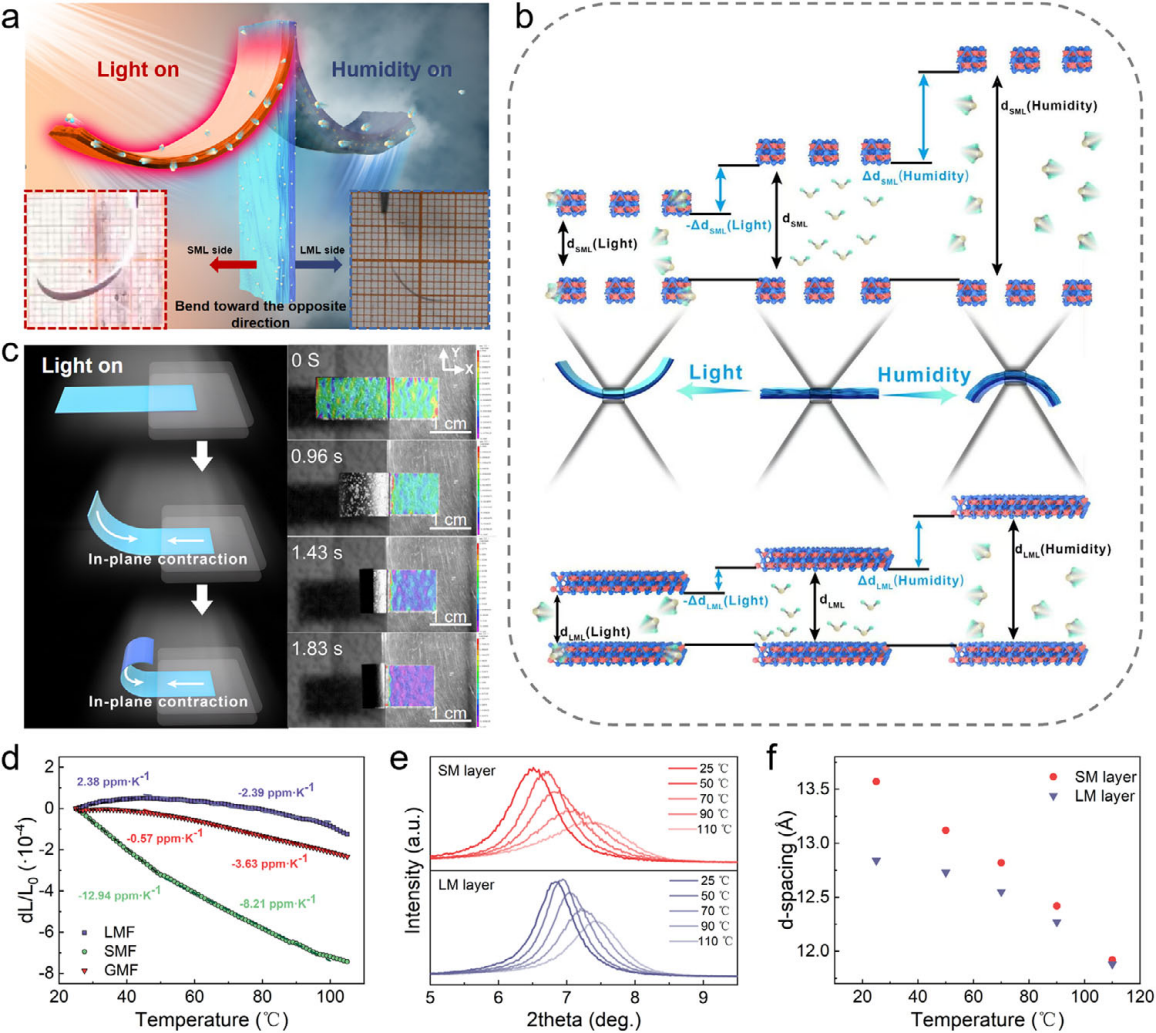
The actuation mechanism analysis of the GMF. a) Schematic diagram of the actuation behavior of the GMF under light irradiation and increasing humidity. b) Schematic diagram of the actuation mechanism of the GMF under light irradiation and increasing humidity. c) Surface strain of the GMF under light irradiation. d) The thermal expansion properties of the LMF, SMF, and GMF. e) XRD patterns of the SM layer and LM layer of GMF under different temperatures. f) Interlayer *d*‐spacing of the SM layer and LM layer of GMF under different temperatures.

Due to its excellent photothermal effect of Ti_3_C_2_T_x_ MXene, this GMF can also achieve light‐induced actuation behavior, with an opposite deformation direction to that of GMF under increasing humidity stimulation (**Figure** **5**a). As shown in Figure 5b, with the ambient RH increases, the larger interlayer free space in SM layer can absorb more water molecules, resulting in a larger interlayer *d*‐spacing increase than that of the LM layer. The interface stress generated by the strain mismatch between LM layer and SM layer causes GMF to bend towards the LM layer side with lower stiffness. For the light‐induced actuation behavior of the GMF, as the temperature increases, water molecules connected to hydrophilic functional groups through hydrogen bonds undergo desorption, reducing the interlayer *d*‐spacing. The asymmetric interlayer *d*‐spacing between SM layer and LM layer implies an asymmetry in the initial water molecules content inside the GMF. The SM layer with higher water molecules content will generate greater compressive stress and deformation during the dehydration process, resulting in the bending deformation of the GMF towards the SM layer side. A noncontact full‐field strain measurement system was used to monitor the deformation process of GMF under light irradiation. Half of the GMF was sandwiched between two pieces of transparent glass (the upper surface is the SM layer), while the other half can deform freely. When the GMF was irradiated, the free part realized bending deformation towards the SM layer side, reaching a maximum deformation of ≈90° within ≈1.8 s. Meanwhile, a gradually increasing X‐direction contraction stress field can be observed on the surface of restricted GMF part, with a maximum strain of −0.008, indicating that the SM layer undergoes shrinkage deformation (Figure 5c).

The thermal expansion performance of LMF, SMF, and GMF were characterized using thermomechanical analysis (TMA). As shown in Figure 5d, LMF, SMF, and GMF all show a trend of contraction with increasing temperature (LMF exhibits abnormal expansion at 25–45 °C, which may be due to the inability of restricted water molecules to deintercalate). The coefficient of thermal expansion (CTE) of SMF (−12.94 ppm K^−1^, 25–50 °C; −8.21 ppm K^−1^, 50–100 °C) is higher than LMF (2.38 ppm K^−1^, 25–45 °C; −2.39 ppm K^−1^, 45–100 °C) and GMF (−0.57 ppm K^−1^, 25–45 °C; −3.63 ppm K^−1^, 45–100 °C), indicating that the asymmetric contraction of SM layer and LM layer leads to the actuation behavior of GMF under light‐induced temperature increase. The XRD of LM layer and SM layer of the GMF were further characterized. As shown in Figure 5e, the (002) diffraction peaks of LM layer and SM layer shift towards larger angles with increasing temperature, indicating that the deintercalation of water molecules leads to a decrease in interlayer *d*‐spacing. As the temperature increases from 25 to 110 °C, the interlayer *d*‐spacing of LM layer and SM layer decreases to almost the same value (≈11.90 Å). However, due to the larger initial interlayer *d*‐spacing of the SM layer, its variation in interlayer *d*‐spacing is larger than that of the LM layer, resulting in the driving deformation of the GMF towards the SM layer side under light‐induced temperature increase.

**Figure 6:**
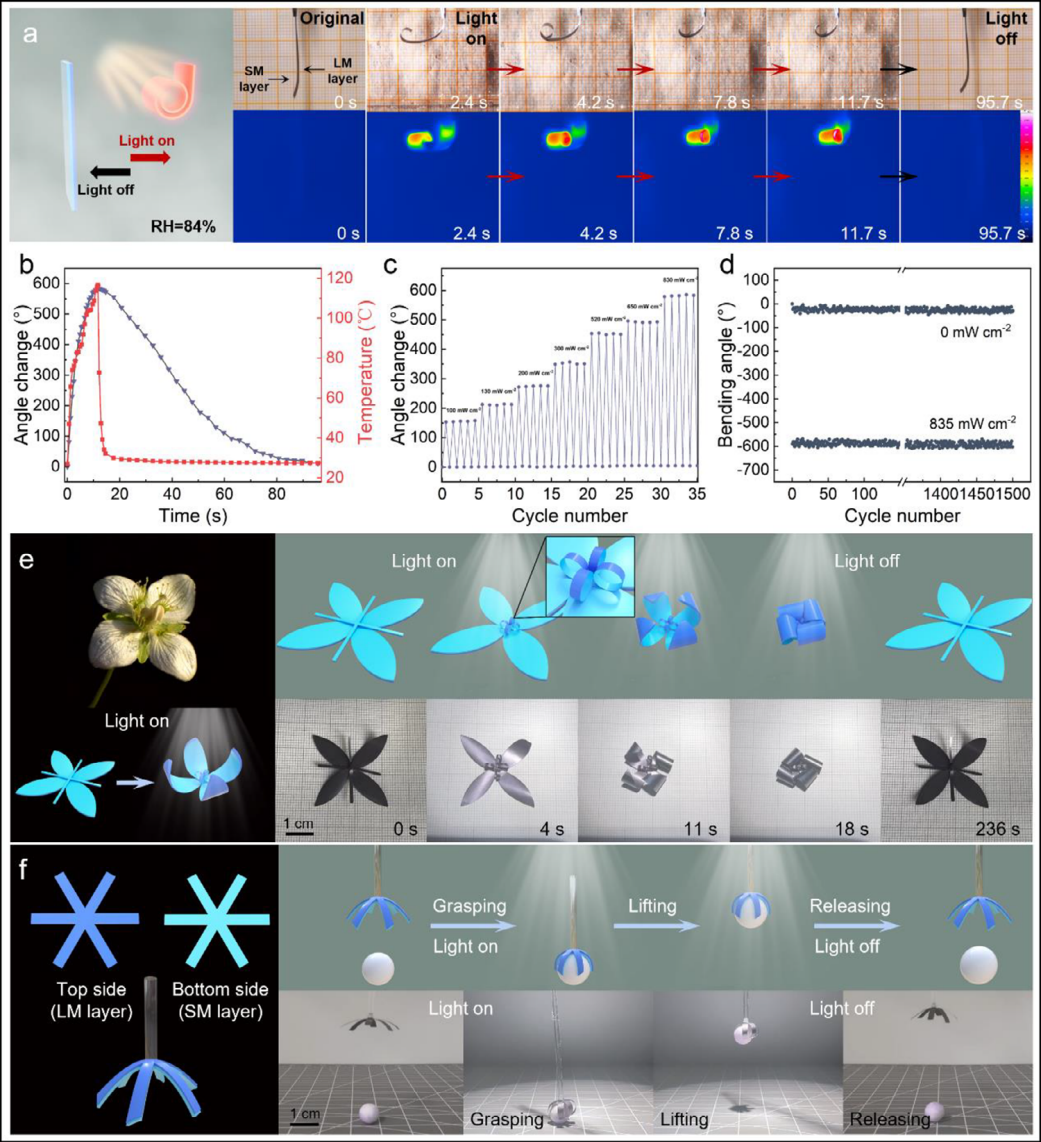
The light‐induced actuation performance of the GMF under high humidity environment. a) Schematic diagram, optical images, and infrared thermal images of the light‐induced actuation behavior of the GMF. b) The real‐time deformation angle and temperature changes of the GMF under light irradiation. c) The actuation performance of the GMF under different light intensities. d) Stability test of the light‐induced actuation behavior of the GMF under 84% RH environment. e) Schematic diagram and optical images of a biomimetic flower based on GMF. f) Schematic and optical images of a light‐controlled gripper based on GMF.

The light‐induced actuation performance of this GMF under a high humidity environment (84% RH) was further studied (Movie S1, Supporting Information). Taking the GMF_84_ as the research object. It is in a nearly flat state at 84% RH. The optical and infrared thermal images of the GMF during its light‐induced actuation process are provided in **Figure** **6**a. When the GMF was irradiated by a light source (835 mW cm^−2^), its temperature rapidly rises and it undergoes bending deformation towards the SM layer side. After ≈11 s of irradiation, the bending angle of the GMF exceeded ≈580°. The actuation performance of this GMF is highly advantageous in thin‐film type soft actuators that do not require polymer assisted deformation (Table S1, Supporting Information). Figure 6b gives the real‐time deformation angle and temperature changes of the GMF under light irradiation. It can be seen that there is a strong correlation between temperature and deformation angle of the GMF during the irradiation stage. When the light irradiation was turned off, the temperature of the GMF rapidly decreases, and the actuator gradually recovers to its initial flat state. The actuation performance of the GMF under different light intensities was further tested by using a stepwise enhancement of light intensity. As shown in Figure 6c, with the increase of light intensity, the deformation angle of the GMF gradually increases. Within five cycles at each light intensity, the deformation of the GMF is stable and reversible, indicating that the deformation of GMF can be precisely controlled by adjusting the light intensity. In addition, this GMF exhibits excellent actuation stability under high humidity environment. As shown in Figure 6d, the maximum bending angle of the GMF remains almost unchanged within 1500 cycles light‐induced actuation under 84% RH environment. The effect of GMF's thickness on its actuation behavior was further investigated. As shown in Figure S6 (Supporting Information), with the increase of thickness, the maximum deformation angle, deformation time, and average deformation speed of the GMF all decrease. The interactive effect of light and humidity stimulation on the actuation behavior of the GMF was also investigated (Figure S7, Supporting Information). When under light radiation, a decrease in humidity will increase the maximum deformation angle of the GMF. When the humidity increases, the light irradiation causes a decrease in the maximum deformation of the GMF. Based on this GMF actuator, a biomimetic flower (Figure 6e; Movie S2, Supporting Information) and a biomimetic gripper (Figure 6f; Movie S3, Supporting Information) were demonstrated. During the recovery process of the GMF actuator, its recovery speed is slower than the deformation speed due to the lack of additional energy input. To improve recovery speed, additional moisture stimulation can be added to the actuating system. As shown in Figure S8 (Supporting Information), using a humidifier to spray water vapor onto the surface of a biomimetic flower can significantly shorten its recovery time. In addition, due to the excellent electric‐thermal conversion ability of Ti_3_C_2_T_x_ MXene, this GMF can also achieve electric‐induced driving deformation. As shown in Figure S9 and Movie S4 (Supporting Information), when a voltage was applied to a U‐shaped GMF, its temperature rapidly increases and undergoes bending deformation towards the SM layer side. Figure S10 (Supporting Information) gives the deformation angle changes and maximum temperature of the GMF under different applied voltages. With the applied voltage increases, the maximum temperature and deformation angle of the GMF also increase. When a voltage of 2.5 V was applied, the deformation angle of the GMF reached up to more than 630°. The stability of the electric‐induced actuation performance of the GMF under high humidity environment (84% RH) was also tested. As shown in the Figure S11 (Supporting Information), after 1500 cycles of electric‐induced driving deformation, the actuation performance of the GMF remains stable.

## 3 Conclusion

In this study, we develop an additive‐free Ti_3_C_2_T_x_ MXene film actuator with large, programmable, stable, and controllable driving deformation. Based on the inherent film‐forming differences of MXene nanosheets of different sizes, additive‐free MXene film with gradient interlayer spacing structures can be constructed through sequential vacuum‐assisted filtration process. The innovative cyclic low‐temperature annealing‐rehydration process endows the MXene film with excellent structural stability and programmable initial shape and deformation range. Based on the differential response to external stimuli caused by the asymmetric interlayer spacing structure of large‐sized MXene layer and small‐sized MXene layer, this additive‐free MXene film can achieve driving deformation under the stimulation of humidity, light, and electricity. The actuation performance of this MXene film is highly advantageous in thin‐film type soft actuators that do not require polymer assisted deformation. In addition, the actuation behavior of this additive‐free MXene film is very stable, even under a high humidity environment (84% RH, more than 1500 cycles of stable light‐induced or electric‐induced driving deformation). This work addresses the trade‐off between the deformation performance and durability of existing MXene‐based actuators, providing a new paradigm for developing soft actuators with large, programmable, stable, and controllable driving deformation, and promoting the development of the next‐generation of soft actuators and related intelligent devices.

## 4 Experimental Section

### Materials

Ti_3_AlC_2_ MAX powder (≥98%; 400 mesh) was acquired from 11 Technology Co., Ltd. (China). Lithium fluoride (LiF, 99.9%) was obtained from Aladdin Chemical Co., Ltd. (China). Hydrochloric acid (HCl, 37%) was purchased from Tianjin Kemiou Chemical Reagent Co., Ltd. (China). All chemicals were used as received.

### Synthesis of the Large‐Sized and Small‐Sized Ti_3_C_2_T_x_ MXene Suspensions

Large‐sized and small‐sized MXene nanosheets were synthesized by the modified LiF/HCl method.^[^ ^42^, ^47^ ^]^ To prepare large‐sized MXene nanosheets, 2 g of MAX powder was added into 42 mL HCl (9 m) containing 3.2 g of LiF. The etching process was carried out for 30 h with 250 rpm stirring at 50 °C. Afterward, the reaction suspension was washed with HCl (1 m) and centrifuged 3–4 times at 3500 rpm to eliminate the excess LiF. The suspension was further washed with deionized water and centrifuged several times at 3500 rpm until the pH of the supernatant was above 6. Then, the sediments were dispersed in deionized water and vigorously shaken with a vortex mixer at 2000 rpm for another 20 min. Finally, the well‐delaminated large‐sized MXene suspension was collected by centrifugation at 1500 rpm for 30 min. The synthesis process of small‐sized MXene nanosheets was similar to that of large‐sized MXene nanosheets. In brief, the reaction temperature and etching time were decreased to 40 °C and 24 h under the same raw material ratio. After being washed and centrifuged with HCl (1 m) and deionized water, the sediments were dispersed in deionized water and sonicated with Ar gas bubbling for 2 h. Then, the suspension was centrifugated at 3500 rpm for 1 h. The small‐sized MXene suspension was obtained from the supernatant. The two MXene suspensions were stored at 4 °C until use.

### Preparation of the Large‐Sized MXene Film and Small‐Sized MXene Film

The large‐sized MXene film and small‐sized MXene film were respectively prepared by vacuum‐assisted filtration of large‐sized MXene suspension and small‐sized MXene suspension, with a suspension concentration of 1.0 mg mL^−1^. The wet films obtained after filtration were then placed in an air environment until completely dry.

### Preparation of the MXene Film with Gradient Structure

The MXene film with gradient structure was obtained through the sequential vacuum‐assisted filtration method. Initially, 10 mL of large‐sized MXene suspension (1.0 mg mL^−1^) was filtered through a nitrocellulose membrane until there was no visible water on the surface. After that, 10 mL of small‐sized MXene suspension (1.0 mg mL^−1^) was added to the surface of the wet large‐sized MXene film, and vacuum‐assisted filtration was continued. Subsequently, the freshly filtered wet MXene film with gradient structure was transferred to a constant humidity box with the specific RH to dry for 36 h, followed by drying in an oven at 60 °C for 10 min. Then, the dried MXene film was again transferred to the specific humidity box for 3 h and rehydration. This process was repeated five times, and the dried MXene film with gradient structure was finally stored in the open air. Here, the RH of the humidity box was adjusted using saturated salt solutions, such as K_2_CO_3_ (43% RH), NaBr (57% RH), NaCl (75% RH), and KCl (84% RH).

### Characterization

The morphology and microstructure of the samples were obtained by scanning electron microscope (SEM, SU5000, Hitachi), transmission electron microscopy (TEM, HT‐7700, Hitachi), and atomic force microscope (AFM, Dimension fastscan, Bruker). X‐ray photoelectron spectroscopy (XPS) was carried out by ESCALAB 250Xi (ThermoFisher). The HRTEM images were obtained by using a high‐resolution transmission electron microscope (JEM‐1400, JEOL). Ultrathin cross sections were prepared by cutting MXene films using a focused ion beam with a TESCAN AMBER system. The XRD spectra were investigated by an X‐ray diffractometer with high‐intensity graphite monochromatized Cu Kα radiation (Rigaku‐TTR3). The thermal expansion properties of the samples were characterized using a thermomechanical analyzer (TMA, Q400EM, TA Instruments). In an air atmosphere, the temperature was raised from 25 to 120 °C at a heating rate of 5 °C min^−1^. The conductivity was measured by a four‐probe resistivity meter (RT‐70 V, Napson). The water contact angle was measured by using a contact angle goniometer (OCA20, Dataphysics). The mechanical properties were tested using a universal testing machine (5944, Instron) at a loading rate of 0.5 mm min^−1^, in which the films were cut into strips (20 mm × 5 mm). The actuation performance of the MXene actuator was recorded by a smartphone. Simulated sunlight irradiation was provided by a Xenon light‐source system (CEL‐HXF300‐T3), and its illumination was measured by an optical power meter (CEL‐NP2000‐2(10)A). The infrared thermal images of the MXene actuator was taken by an infrared camera (VarioCAM HD head 880, InfraTec) to record the real‐time surface temperatures. Minor strains on the film surface during the deformation were characterized using a Video Image Correlation in 3D (VIC 3D) system. Prior to testing, black matte paint was sprayed onto the film surface, followed by white paint, to record the position.

## Conflict of Interest

The authors declare no conflict of interest.

## Supporting information



Supporting Information

Supplemental Movie 1

Supplemental Movie 2

Supplemental Movie 3

Supplemental Movie 4

## Data Availability

The data that support the findings of this study are available from the corresponding author upon reasonable request.

## References

[1] C. Ni , D. Chen , X. Wen , B. Jin , Y. He , T. Xie , Q. Zhao , Nat. Commun. 2023, 14, 7672. 37996451 10.1038/s41467-023-43576-6 PMC10667353

[2] S. Kim , Y.‐H. Hsiao , Y. Lee , W. Zhu , Z. Ren , F. Niroui , Y. Chen , Sci. Rob. 2023, 8, adf4278. 10.1126/scirobotics.adf4278 36921017

[3] X. Li , Y. Du , X. Pan , C. Xiao , X. Ding , K. Zheng , X. Liu , L. Chen , Y. Gong , M. Xue , X. Tian , X. Zhang , Adv. Mater. 2025, 37, 2416991. 10.1002/adma.202416991 39955736

[4] Y. Hu , L. Yang , Q. Yan , Q. Ji , L. Chang , C. Zhang , J. Yan , R. Wang , L. Zhang , G. Wu , J. Sun , B. Zi , W. Chen , Y. Wu , ACS Nano 2021, 15, 5294. 33650851 10.1021/acsnano.0c10797

[5] G. Tang , X. Zhao , S. Liu , D. Mei , C. Zhao , L. Li , Y. Wang , Adv. Funct. Mater. 2025, 35, 2412254.

[6] D. Podbevšek , Y. Jung , M. K. Khan , H. Yu , R. S. Tu , X. Chen , Nat. Commun. 2024, 15, 8287. 39333569 10.1038/s41467-024-52715-6 PMC11436739

[7] Y. Wu , S. Zhang , Y. Yang , Z. Li , Y. Wei , Y. Ji , Sci. Adv. 2022, 8, abo6021. 10.1126/sciadv.abo6021 PMC9232107 35749490

[8] C. Wu , J. Li , Q. Zhang , H. Kang , Z. Xie , Z. Cheng , Q. Tao , D. Zhang , Y. Liu , Adv. Funct. Mater. 2025, 35, 2419520.

[9] M. Wang , L. Zhou , W. Deng , Y. Hou , W. He , L. Yu , H. Sun , L. Ren , X. Hou , ACS Nano 2022, 16, 2672. 35040625 10.1021/acsnano.1c09477

[10] X.‐J. Luo , L. Li , H.‐B. Zhang , S. Zhao , Y. Zhang , W. Chen , Z.‐Z. Yu , ACS Appl. Mater. Interfaces 2021, 13, 45833. 34520189 10.1021/acsami.1c11056

[11] J. Li , B. Liu , W. Chen , S. Zhang , J. Deng , Y. Liu , Adv. Funct. Mater. 2025, 35, 2422499.

[12] Z. Che , X. Wan , J. Xu , C. Duan , T. Zheng , J. Chen , Nat. Commun. 2024, 15, 1873. 38472193 10.1038/s41467-024-45915-7 PMC10933441

[13] L. Mao , P. Yang , C. Tian , X. Shen , F. Wang , H. Zhang , X. Meng , H. Xie , Nat. Commun. 2024, 15, 3759. 38704384 10.1038/s41467-024-48058-x PMC11069526

[14] T. Zhang , G. Li , H. Ren , L. Yang , X. Yang , R. Tan , Y. Tang , D. Guo , H. Zhao , W. Shang , Y. Shen , Nat. Commun. 2024, 15, 10874. 39738028 10.1038/s41467-024-55199-6 PMC11685957

[15] J. Xiong , X. Li , Z. He , Y. Shi , T. Pan , G. Zhu , D. Lu , H. Xin , Light: Sci. Appl. 2024, 13, 55. 38403642 10.1038/s41377-024-01405-5 PMC10894875

[16] S. Chen , J. Ciou , F. Yu , J. Chen , J. Lv , P. S. Lee , Adv. Mater. 2022, 34, 2200660. 10.1002/adma.202200660 35584538

[17] T. Dai , Y. Liu , D. Rong , M. Wang , Z. Qi , Y. Zhao , X. Wang , Q. Yang , L. Wei , M. Chen , Adv. Funct. Mater. 2024, 34, 2400459.

[18] L. Yang , J. Cui , L. Zhang , X. Xu , X. Chen , D. Sun , Adv. Funct. Mater. 2021, 31, 2101378.

[19] J. Cao , Z. Zhou , Q. Song , K. Chen , G. Su , T. Zhou , Z. Zheng , C. Lu , X. Zhang , ACS Nano 2020, 14, 7055. 32441915 10.1021/acsnano.0c01779

[20] B. Liu , Z. Ling , J. Du , J. Qiu , Small 2025, 21, 2409341. 10.1002/smll.202409341 40091377

[21] L. Xu , Q. Peng , X. Zhao , P. Li , J. Xu , X. He , ACS Appl. Mater. Interfaces 2020, 12, 40711. 32805842 10.1021/acsami.0c14222

[22] B. Wu , M. Si , L. Hua , D. Zhang , W. Li , C. Zhao , W. Lu , T. Chen , Adv. Mater. 2024, 36, 2401659. 10.1002/adma.202401659 38533903

[23] L. Xu , F. Xue , H. Zheng , Q. Ji , C. Qiu , Z. Chen , X. Zhao , P. Li , Y. Hu , Q. Peng , X. He , Nano Energy 2022, 103, 107848.

[24] X. Yang , L. Lan , L. Li , J. Yu , X. Liu , Y. Tao , Q.‐H. Yang , P. Naumov , H. Zhang , Nat. Commun. 2023, 14, 3627. 37336878 10.1038/s41467-023-39162-5 PMC10279756

[25] G. Li , B. C. Wyatt , F. Song , C. Yu , Z. Wu , X. Xie , B. Anasori , N. Zhang , Adv. Funct. Mater. 2021, 31, 2105043.

[26] V. H. Nguyen , R. Tabassian , S. Oh , S. Nam , M. Mahato , P. Thangasamy , A. Rajabi‐Abhari , W. Hwang , A. K. Taseer , I. Oh , Adv. Funct. Mater. 2020, 30, 1909504.

[27] D. Pang , M. Alhabeb , X. Mu , Y. Dall'Agnese , Y. Gogotsi , Y. Gao , Nano Lett. 2019, 19, 7443. 31536705 10.1021/acs.nanolett.9b03147

[28] Q. Gao , J. Come , M. Naguib , S. Jesse , Y. Gogotsi , N. Balke , Faraday Discuss. 2017, 199, 393. 28429016 10.1039/c6fd00251j

[29] A. Di , C. Wang , Y. Wang , H. He , W. Deng , P. Stiernet , L. Bergström , J. Yuan , M. Zhang , Chem. Sci. 2025, 16, 2191. 39664811 10.1039/d4sc04935g PMC11629778

[30] S. Chen , S. F. Tan , H. Singh , L. Liu , M. Etienne , P. S. Lee , Adv. Mater. 2024, 36, 2307045. 10.1002/adma.202307045 37787743

[31] W. Wang , T. Cai , L. Tang , J. Zhang , C. Du , J. Tang , Y. Yang , L. Yin , H. Kang , Z. Fan , ACS Appl. Nano Mater. 2023, 6, 18721.

[32] Q. Zhang , H. Lai , R. Fan , P. Ji , X. Fu , H. Li , ACS Nano 2021, 15, 5249. 33617227 10.1021/acsnano.0c10671

[33] D. Qu , Y. Jian , L. Guo , C. Su , N. Tang , X. Zhang , W. Hu , Z. Wang , Z. Zhao , P. Zhong , P. Li , T. Du , H. Haick , W. Wu , Nano Micro Lett. 2021, 13, 188. 10.1007/s40820-021-00705-4 PMC8418585 34482476

[34] X. Shen , R. Hai , X. Wang , Y. Li , Y. Wang , F. Yu , J. Ma , J. Mater. Chem. A 2020, 8, 19309.

[35] S. Zheng , C. (John) Zhang , F. Zhou , Y. Dong , X. Shi , V. Nicolosi , Z.‐S. Wu , X. Bao , J. Mater. Chem. A 2019, 7, 9478.

[36] W. Li , M. Sang , S. Liu , B. Wang , X. Cao , G. Liu , X. Gong , L. Hao , S. Xuan , Composites, Part B 2022, 238, 109880.

[37] W. Li , C. Lou , S. Liu , Q. Ma , G. Liao , K. C. Leung , X. Gong , H. Ma , S. Xuan , Adv. Funct. Mater. 2025, 35, 2414733.

[38] S. Xue , Z. Shi , Z. Wang , H. Tan , F. Gao , Z. Zhang , Z. Ye , S. Nian , T. Han , J. Zhang , Z. Zhao , B. Z. Tang , Q. Zhang , Nat. Commun. 2024, 15, 10084. 39572542 10.1038/s41467-024-54386-9 PMC11582805

[39] S. Lin , S. Ma , K. Chen , Y. Zhang , Z. Lin , Y. Liang , L. Ren , Chem. Eng. J. 2024, 495, 153294.

[40] Z. Zhang , Z. Li , W. Yuan , Small 2025, 21, 2501164. 10.1002/smll.202501164 40195803

[41] Y. Zhang , X. Zhou , L. Liu , S. Wang , Y. Zhang , M. Wu , Z. Lu , Z. Ming , J. Tao , J. Xiong , Adv. Mater. 2024, 36, 2404696. 10.1002/adma.202404696 38923035

[42] X. Zhang , X. Liu , Q. Liu , Y. Feng , S. Qiu , T. Wang , H. Xu , H. Li , L. Yin , H. Kang , Z. Fan , Adv. Sci. 2024, 11, 2309171. 10.1002/advs.202309171 PMC11186054 38582527

[43] Q. Zhang , R. Fan , W. Cheng , P. Ji , J. Sheng , Q. Liao , H. Lai , X. Fu , C. Zhang , H. Li , Adv. Sci. 2022, 9, 2202748. 10.1002/advs.202202748 PMC9534978 35975421

[44] J. Zhang , N. Kong , S. Uzun , A. Levitt , S. Seyedin , P. A. Lynch , S. Qin , M. Han , W. Yang , J. Liu , X. Wang , Y. Gogotsi , J. M. Razal , Adv. Mater. 2020, 32, 2001093. 10.1002/adma.202001093 32309891

[45] M. C. Biesinger , L. W. M. Lau , A. R. Gerson , R. S. C. Smart , Appl. Surf. Sci. 2010, 257, 887.

[46] S. Wan , X. Li , Y. Chen , N. Liu , S. Wang , Y. Du , Z. Xu , X. Deng , S. Dou , L. Jiang , Q. Cheng , Nat. Commun. 2022, 13, 7340. 36446803 10.1038/s41467-022-35226-0 PMC9708659

[47] J. Wang , Y. Liu , Y. Yang , J. Wang , H. Kang , H. Yang , D. Zhang , Z. Cheng , Z. Xie , H. Tan , Z. Fan , Matter 2022, 5, 1042.

[48] A. Kamal , B. Li , S. Luo , I. Kinloch , L. Zheng , K. Liao , Nano Mater. Sci. 2025, S2589965125000285.

[49] H. Chen , Y. Wen , Y. Qi , Q. Zhao , L. Qu , C. Li , Adv. Funct. Mater. 2020, 30, 1906996.

