# Supplementary material for: Additive‐Free Ti3C2Tx MXene Actuator with Large Deformation, Programmability, and High‐Humidity Stability via Precise Interlayer Spacing Control Engineering
Source: Adv Sci (Weinh). 2025 Aug 25;13(15):e10243. doi: 10.1002/advs.202510243 (PMC13042935; doi:10.1002/advs.202510243)
Supplement: Supplementary file 1 — Supporting Information [file ADVS-13-e10243-s001.docx]

Supporting Information

**Additive-Free Ti_3_C_2_T_x_ MXene Actuator with Large Deformation, Programmability, and High-Humidity Stability via Precise Interlayer Spacing Control Engineering**

*Haowen Zheng, Liangliang Xu, Qian Yan, Zonglin Liu, He Chen, Huanxin Lian, Yunxiang Chen, Teng Fei, Yiming Hu, Fuhua Xue, Xu Zhao, Cong Zhang*, Qingyu Peng*, Xiaodong He**

**Supplemental Figures**


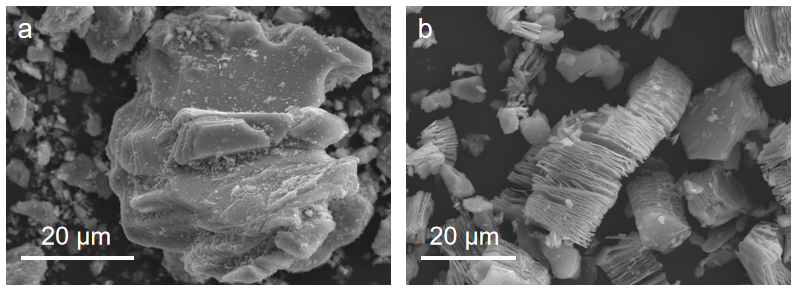


**Figure S1** SEM images of (a) Ti_3_AlC_2_ powder with compact layered structure and (b) multilayered Ti_3_C_2_T_x_ powder with a typical accordion-like structure.


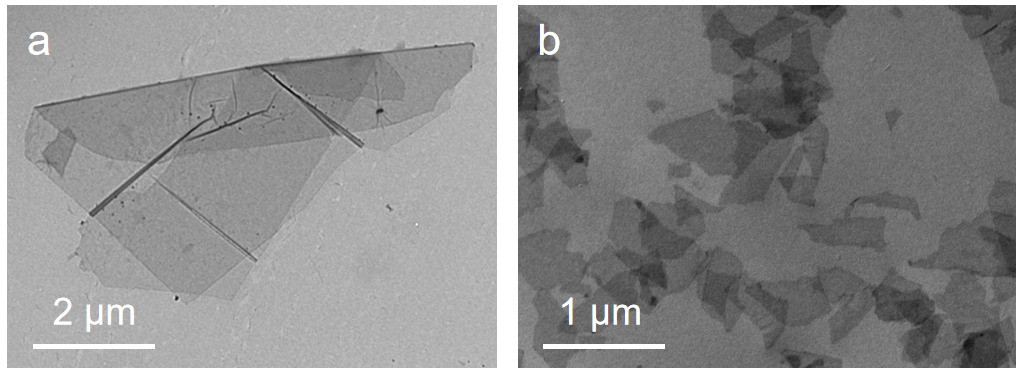


**Figure S2** TEM images of (a) LM nanosheets and (b) SM nanosheets.


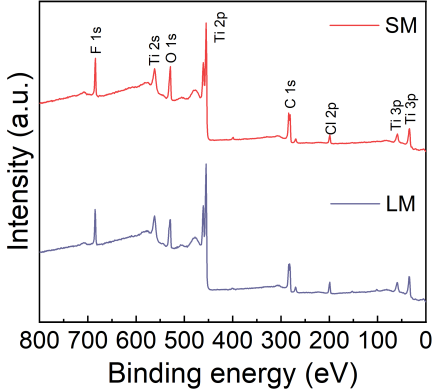


**Figure S3** XPS spectra of SM and LM.


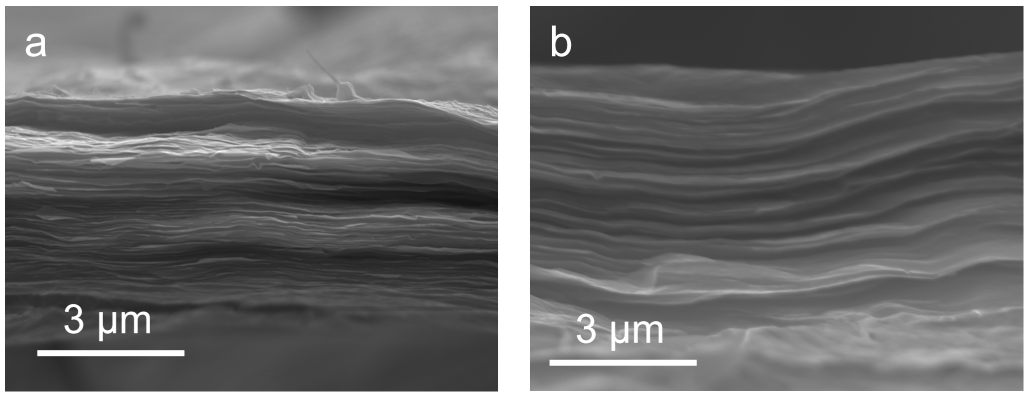


**Figure S4** Cross-sectional SEM images of (a) LMF and (b) SMF.


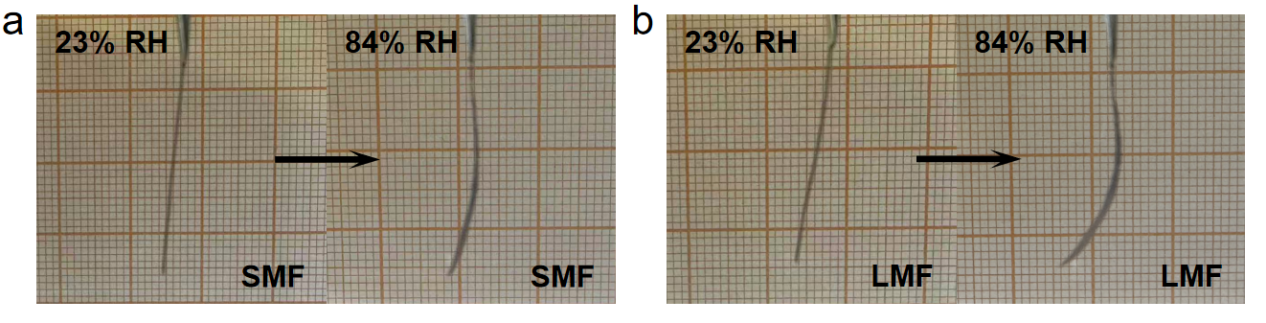


**Figure S5** Optical images of (a) SMF and (b) LMF at 23% and 84% RH environments.


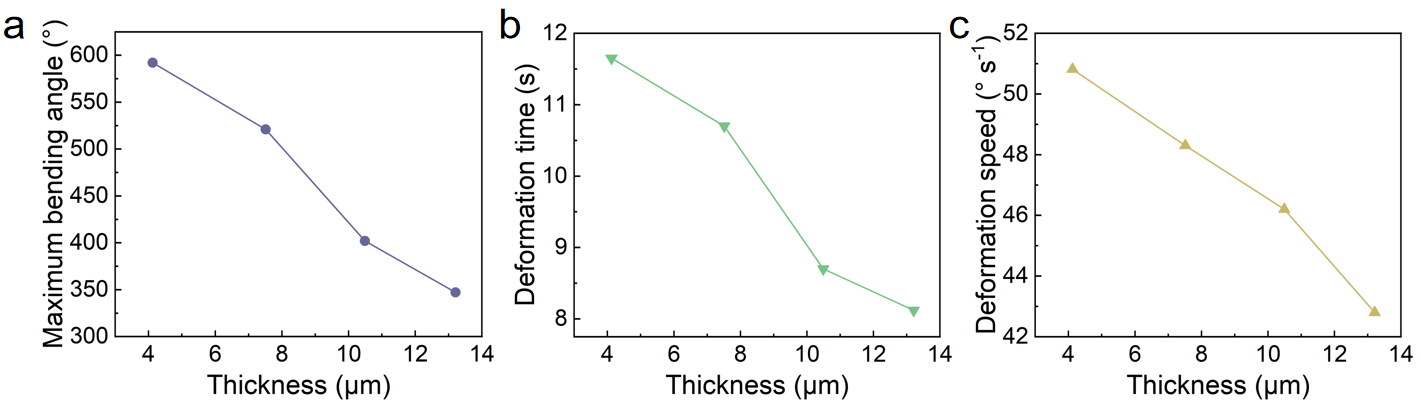


**Figure S6** (a) Maximum bending angle, (b) deformation time, and (c) average deformation speed of the GMF with different thickness.


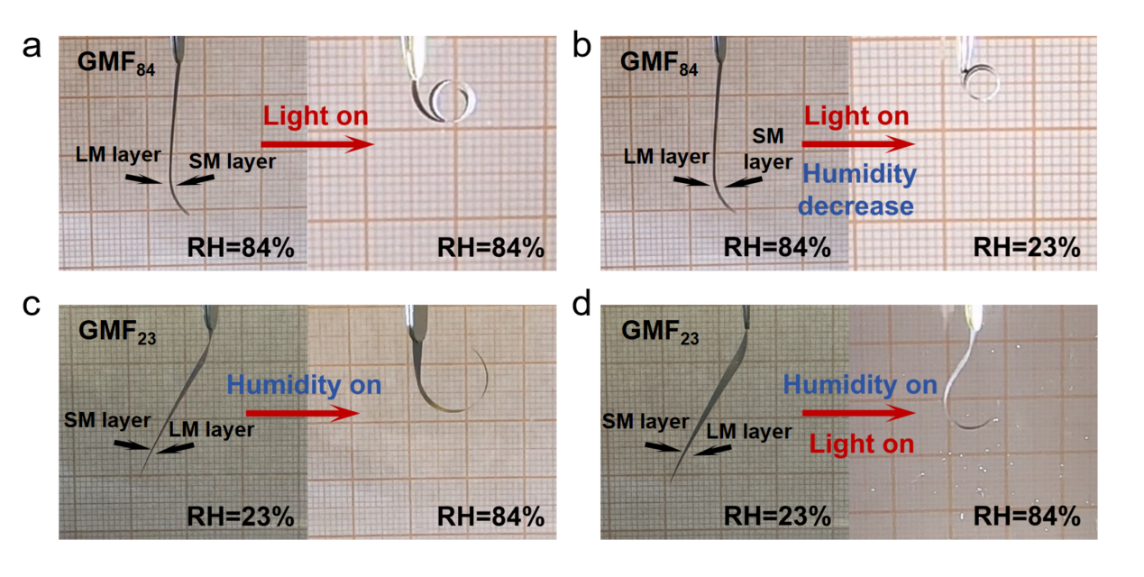


**Figure S7.** (a) Optical images of the GMF under light irradiation only and (b) light irradiation with decreased humidity. (c) Optical images of the GMF under increased humidity only and (d) increased humidity with light irradiation.


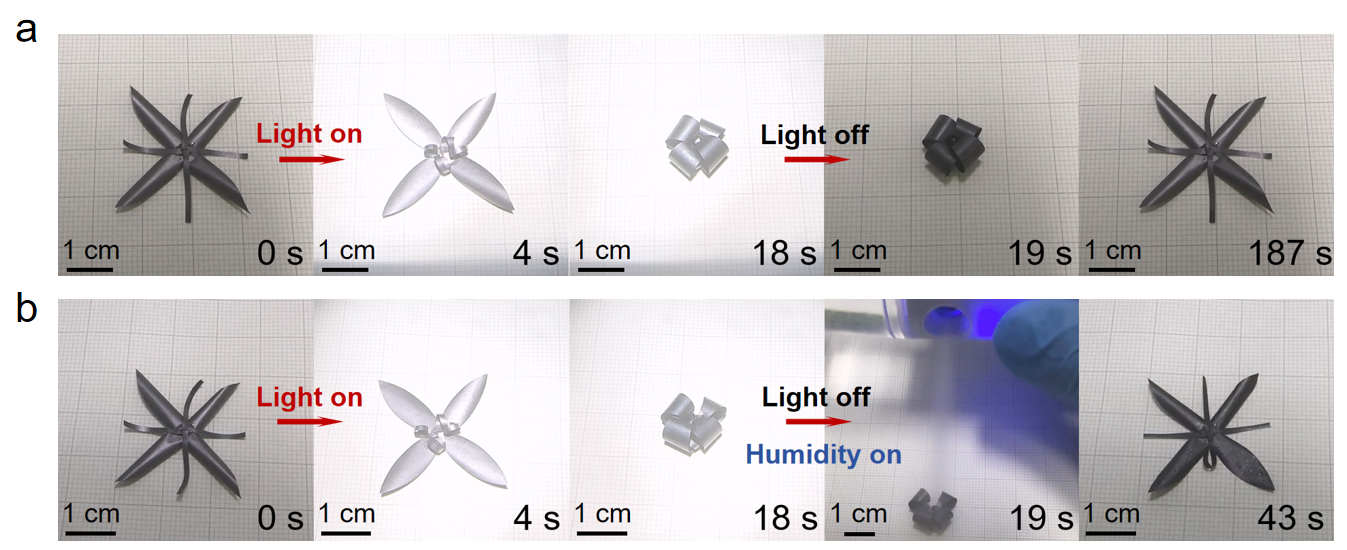


**Figure S8.** (a) Optical images of the deformation process of a biomimetic flower under constant humidity. (b) Optical images of the deformation process of a biomimetic flower under increased humidity during the recovery process.


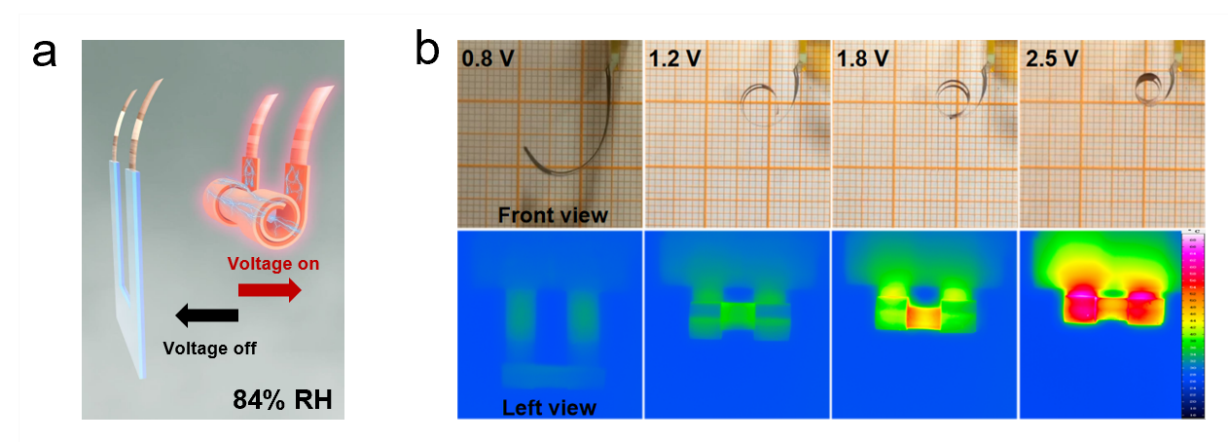


**Figure S9** (a) Schematic diagram of the electric-driven actuation of the GMF. (b) Optical and infrared thermal images of the GMF under different applied voltages.

**
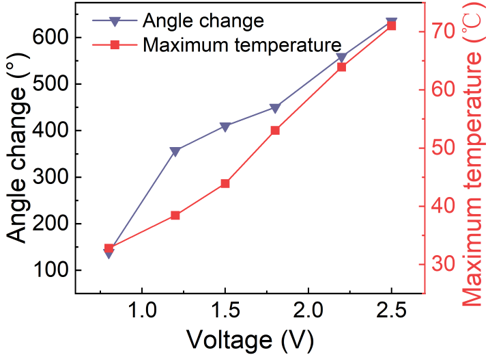
**

**Figure S10** Deformation angle and maximum temperature of the GMF under different applied voltages.


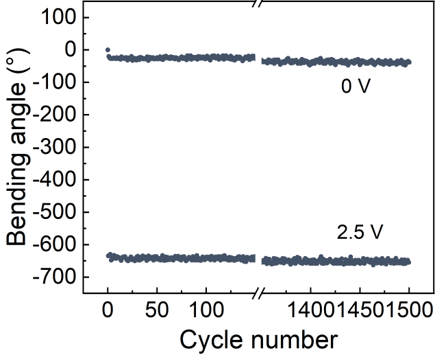


**Figure S11** Stability test of the electric-driven actuation behavior of the GMF under 84% RH environment

**Supplemental Table**

**Table S1** Comparison of the actuation performance of this MXene actuator with other typical MXene-based actuators.

| **Materials** | **The types of stimulus source** | **Maximum bending angle change** | **Electrical conductivity/Sheet resistances** | **Programmability** | **Cycle stability** | **Reference** |
| --- | --- | --- | --- | --- | --- | --- |
| Uniform MXene film | Humidity | 155°  ( ΔRH = 65%) | 3120 S cm^-1^ | No | ΔRH = 65%, bending angel ≈ 105°,  10 cycles | 1 |
| Polydopamine-MXene/Bacterial Cellulose | Humidity | 176°  (ΔRH = 40%) | 2848 S cm^-1^ | No | After 60 days in atmosphere,  bending angel ≈ 175°, 50 cycles | 2 |
| Polydopamine/rGO/MXene | Humidity | 148°  (ΔRH = 45%) | 3693 S cm^-1^ | No | ΔRH = 65%, bending angel ≈ 148°,  60 cycles | 3 |
| Methylcellulose-MXene/TPU/CMF | Electricity | ~ 50°  (± 0.93 V) | 36.4 S cm^-1^ | No | RH = 65%, 79.8% initial displacement,  5000 cycles | 4 |
| PTFE/MXene/PI | Electricity | 122°  (6 V) | 1860 S cm^-1^ | No | Atmosphere, 6 V, bending angel ≈ 122°,  10 cycles | 5 |
| Tetrabutylammonium-MXene/TPU/CMF | Electricity | ~ 80°  (± 0.85 V) | 179 S cm^-1^ | No | RH = 65%, 102.6% initial displacement,  10000 cycles | 6 |
| MXene-CNF/PDMS | Light | 50°  (200 mW cm^-2^) | / | No | Atmosphere, 200 mW cm^-2^,  bending angel ≈ 50°, 100 cycles | 7 |
| MCPM/PET  (MCPM:MXene/Cellulose/  Polystyrene sulfonic acid) | Humidity/light | 102°  ( ΔRH = 77%) | 9.31 S m^-1^ | No | RH = 80%, 24 mW cm^-2^,  bending angel ≈ 115°, 12 cycles | 8 |
| MPDMS/MXene/PTFE  (MPMS:NdFeB/PDMS) | Electricity/  magnetic field | 353°  (3 V) | 2100 S cm^-1^ | No | / | 9 |
| MXene/VO_2_@PMMA | Humidity/light/  electricity | 127°  (1 solar intensity) | / | No | Atmosphere, displacement ≈ 2 mm,  10 cycles | 10 |
| MXene/PDMS | Light/electricity/  heat | 250°  (300 mW cm^-2^) | / | No | Atmosphere, 300 mW cm^-2^,  bending angel ≈ 250°, 100 cycles | 11 |
| CMPC/PTFE  (CMPC:Cellulose/MXene/PEDOT:PSS) | Humidity/light/  electricity | 360°  ( ΔRH = 4%) | 7.2 Ω sq^-1^ | No | RH=32%, bending angel ≈ 360°, 500 cycles | 12 |
| MXCC/PC  (MXCC:MXene/Cellulose) | Humidity/light/  electricity | 175°  (ΔRH = 85%) | / | Yes | Atmosphere, 50 mW cm^-2^,  100% initial weight，1000 cycles | 13 |
| MXene/Low-density PE | Near-infrared light/electricity/heat | 916°  (1.7 V) | / | Yes | Atmosphere, 83 mW cm^-2^,  bending angel ≈ 350°, 500 cycles；  Atmosphere, 1.4 V,  bending angel change ≈ 490°, 500 cycles | 14 |
| **MXene film with gradient structure** | **Humidity/light/**  **electricity** | **523°(ΔRH = 61%)**  **586°(835 mW cm^-2^)**  **635°(2.5 V)** | **7453 S cm^-1^** | **Yes** | **RH = 84%, 835 mW cm^-2^**  **bending angel change ≈ 580°, 1500 cycles；**  **RH = 84%, 2.5 V**  **bending angel change ≈ 630°, 1500 cycles** | **This work** |

**Supplemental Videos**

**Movie S1** Light-induced actuation behavior of the GMF under 84% RH.

**Movie S2** A light-driven biomimetic flower.

**Movie S3** A light-driven biomimetic gripper.

**Movie S4** Electric-induced actuation behavior of the GMF under 84% RH.

**References**

[1] J. Wang, Y. Liu, Z. Cheng, Z. Xie, L. Yin, W. Wang, Y. Song, H. Zhang, Y. Wang, Z. Fan, *Angew. Chem. Int. Ed.* **2020**, *59*, 14029.

[2] L. Yang, J. Cui, L. Zhang, X. Xu, X. Chen, D. Sun, *Adv. Funct. Mater.* **2021**, *31*, 2101378.

[3] L. Yang, L. Zhang, J. Cui, D. Sun, *J. Mater. Chem. A* **2022**, *10*, 15785.

[4] S. Chen, J. Ciou, F. Yu, J. Chen, J. Lv, P. S. Lee, *Adv. Mater.* **2022**, *34*, 2200660.

[5] M. Sang, G. Liu, S. Liu, Y. Wu, S. Xuan, S. Wang, S. Xuan, W. Jiang, X. Gong, *Chem. Eng. J.* **2021**, *414*, 128883.

[6] S. Chen, S. F. Tan, H. Singh, L. Liu, M. Etienne, P. S. Lee, *Adv. Mater.* **2024**, *36*, 2307045.

[7] T. Dai, Y. Liu, D. Rong, M. Wang, Z. Qi, Y. Zhao, X. Wang, Q. Yang, L. Wei, M. Chen, *Adv. Funct. Mater.* **2024**, *34*, 2400459.

[8] P. Li, N. Su, Z. Wang, J. Qiu, *ACS Nano* **2021**, *15*, 16811.

[9] W. Li, M. Sang, S. Liu, B. Wang, X. Cao, G. Liu, X. Gong, L. Hao, S. Xuan, *Composites, Part B* **2022**, *238*, 109880.

[10] B. Liu, Z. Ling, J. Du, J. Qiu, *Small* **2025**, *21*, 2409341.

[11] L. Xu, H. Zheng, F. Xue, Q. Ji, C. Qiu, Q. Yan, R. Ding, X. Zhao, Y. Hu, Q. Peng, X. He, *Chemical Engineering Journal* **2023**, *463*, 142392.

[12] Y. Li, J. Wang, L. Huang, L. Chen, H. Gao, Y. Ni, Q. Zheng, *ACS Sustainable Chem. Eng.* **2022**, *10*, 6414.

[13] G. Cai, J.-H. Ciou, Y. Liu, Y. Jiang, P. S. Lee, *Sci. Adv.* **2019**, *5*, eaaw7956.

[14] X.-J. Luo, L. Li, H.-B. Zhang, S. Zhao, Y. Zhang, W. Chen, Z.-Z. Yu, *ACS Appl. Mater. Interfaces* **2021**, *13*, 45833.
